# Outcomes Among Patients Referred to Outpatient Rehabilitation Clinics After COVID-19 diagnosis — United States, January 2020–March 2021

**DOI:** 10.15585/mmwr.mm7027a2

**Published:** 2021-07-09

**Authors:** Jessica S. Rogers-Brown, Valentine Wanga, Catherine Okoro, Diane Brozowsky, Alan Evans, David Hopwood, Jennifer R. Cope, Brendan R. Jackson, Dena Bushman, Alfonso C. Hernandez-Romieu, Robert A. Bonacci, Tim McLeod, Jennifer R. Chevinsky, Alyson B. Goodman, Meredith G. Dixson, Caitlyn Lufty, Julie Rushmore, Emily Koumans, Sapna Bamrah Morris, William Thompson

**Affiliations:** ^1^CDC COVID-19 Response Team; ^2^Division of Viral Hepatitis, National Center for HIV/AIDS, Viral Hepatitis, STD, and TB Prevention, CDC; ^3^Epidemic Intelligence Service, CDC; ^4^Division of Human Development and Disability, National Center on Birth Defects and Developmental Disabilities, CDC; ^5^Select Medical, Mechanicsburg, Pennsylvania.

As of June 30, 2021, 33.5 million persons in the United States had received a diagnosis of COVID-19 (*1*). Although most patients infected with SARS-CoV-2, the virus that causes COVID-19, recover within a few weeks, some experience post–COVID-19 conditions. These range from new or returning to ongoing health problems that can continue beyond 4 weeks. Persons who were asymptomatic at the time of infection can also experience post–COVID-19 conditions. Data on post–COVID-19 conditions are emerging and information on rehabilitation needs among persons recovering from COVID-19 is limited. Using data acquired during January 2020–March 2021 from Select Medical* outpatient rehabilitation clinics, CDC compared patient-reported measures of health, physical endurance, and health care use between patients who had recovered from COVID-19 (post–COVID-19 patients) and patients needing rehabilitation because of a current or previous diagnosis of a neoplasm (cancer) who had not experienced COVID-19 (control patients). All patients had been referred to outpatient rehabilitation. Compared with control patients, post–COVID-19 patients had higher age- and sex-adjusted odds of reporting worse physical health (adjusted odds ratio [aOR] = 1.8), pain (aOR = 2.3), and difficulty with physical activities (aOR = 1.6). Post–COVID-19 patients also had worse physical endurance, measured by the 6-minute walk test^†^ (6MWT) (p<0.001) compared with control patients. Among patients referred to outpatient rehabilitation, those recovering from COVID-19 had poorer physical health and functional status than those who had cancer, or were recovering from cancer but not COVID-19. Patients recovering from COVID-19 might need additional clinical support, including tailored physical and mental health rehabilitation services.

Data were obtained from electronic health records (EHRs) of patients referred to Select Medical’s outpatient rehabilitation clinics during January 2020–March 2021. Epidemiologic, clinical, and functional data from 1,295 post–COVID-19 patients and 2,395 control patients were examined. Post-COVID-19 patients were defined as those who were referred to a Select Medical facility for post–COVID-19 physical rehabilitation. Control patients, defined as those needing rehabilitation for a current or previous diagnosis of cancer with no history of an *International Classification of Diseases, Tenth Revision* (ICD-10) COVID-19 diagnosis code,^§^ were referred to a Select Medical cancer rehabilitation program. This control population was chosen because patients in this group completed the same initial evaluations as patients referred for post–COVID-19 rehabilitation. Information on type of cancer or interval since diagnosis was not available. Patient data were collected from EHRs and initial clinical evaluation, which included self-reported health measures and a 6MWT. At intake, self-reported measures and clinical evaluations were administered for health, physical endurance, and health care use.

Using validated scales, CDC assessed patients’ mental and physical health, functional health, social participation ability, applied cognition, and physical endurance with Patient-Reported Outcomes Measurement Information System (PROMIS) Global Health (version 1.2; National Institutes of Health), PROMIS Physical Function, PROMIS Ability,^¶^ Quality of Life in Neurologic Disorders (Neuro-QoL),** and the 6MWT,^††^ respectively. For self-reported item-level data, five-point Likert scales were recoded to proportions. T-scores were computed for composite measures of physical and mental health, social participation ability, and applied cognition, where the summed raw scores were converted to T-scores based on standardized scoring tables; T-scores were designed to have a mean of 50 and a standard deviation (SD) of 10 for the general adult population Logistic regression analysis, adjusted for age and sex, was used to examine differences in patient-reported measures of health, physical endurance, and health care use between post–COVID-19 and control patients.^§§^ All analyses were conducted using SAS (version 9.4; SAS Institute). This activity was reviewed by CDC and was conducted consistent with applicable federal law and CDC policy.^¶¶^

Post–COVID-19 patients referred for rehabilitation services differed from control patients by several characteristics, including sex, age, race, ethnicity, employment status, health insurance coverage, and U.S. Census region (Table 1). Compared with control patients, post–COVID-19 patients were more likely to be male, younger, in the labor force, insured by a commercial plan or a worker’s compensation plan, and less likely to be covered by Medicaid or Medicare (Table 1). Post–COVID-19 patients were more likely to have received a diagnosis of generalized muscle weakness or fatigue (72.7% versus 42.3%) and patient-reported symptoms of generalized muscle weakness, malaise, and fatigue (69.0% versus 59.7%) (Table 2).

**TABLE 1:** Baseline characteristics of post–COVID-19 patients and control patients* who received care in outpatient rehabilitation clinics — United States,**^†^** January 2020–March 2021

Characteristic	No. (%)		p-value^§^
	Post–COVID-19 patients (n = 1,295)	Control patients (n = 2,395)	
**Sex**			
Male	560 (43.2)	610 (25.5)	<0.001
Female	735 (56.8)	1,785 (74.5)	
**Age, median (IQR), yrs**	56 (44–65)	61 (51–70)	<0.001
**Age group, yrs**			
18–39	233 (18.0)	155 (6.5)	<0.001
40–49	197 (15.2)	325 (13.6)	
50–59	355 (27.4)	611 (25.5)	
60–69	282 (21.8)	665 (27.8)	
70–79	163 (12.6)	499 (20.8)	
≥80	65 (5.0)	140 (5.8)	
**Race^¶^**			
White	320 (24.7)	814 (34.0)	<0.001
Black or African American	101 (7.8)	173 (7.2)	
Other	36 (2.8)	51 (2.1)	
Missing	838 (64.7)	1,357 (56.7)	
**Ethnicity**			
Hispanic or Latino	92 (7.1)	75 (3.1)	<0.001
Missing	1,203 (92.9)	2,320 (96.9)	
**Marital status**			
Married	624 (48.2)	1,209 (50.5)	0.122
Single	250 (19.3)	413 (17.2)	
Other (not specified)	81 (6.3)	119 (5.0)	
Missing	340 (26.3)	654 (27.3)	
**Employment status**			
In labor force	271 (20.9)	415 (17.3)	<0.001
Not in labor force	48 (3.7)	488 (20.4)	
Missing	976 (75.4)	1,492 (62.3)	
**Health insurance coverage**			
Medicaid/Medicare	433 (33.4)	1,074 (44.8)	<0.001
Private/Commercial	746 (57.6)	1,291 (53.9)	
Other**	116 (9.0)	30 (1.3)	
**U.S. Census region**			
Midwest	230 (17.8)	380 (15.9)	<0.001
Northeast	410 (31.7)	438 (18.3)	
South	568 (43.9)	1,304 (54.4)	
West	86 (6.6)	273 (11.4)	
Missing	1 (<0.01)	0 (—)	

**Abbreviations: **ICD-10 = *International Classification of Diseases, Tenth Revision*; IQR = interquartile range.

* Post–COVID-19 patients in this analysis were patients referred to Select Medical’s Recovery and Reconditioning program that includes post–COVID-19 care. In addition, patient history of COVID-19 was assessed to validate that each patient had either 1) an ICD-10 code for COVID-19 or 2) clinical notes documenting COVID-19 history. Control patients were patients referred for cancer rehabilitation and confirmed with no history of COVID-19 diagnoses by ICD-10 code in the same network and time frame.

^†^ Select Medical’s Recovery and Reconditioning clinics are located in Alabama, Alaska, Arizona, California, Colorado, Connecticut, Delaware, Florida, Georgia, Illinois, Indiana, Iowa, Kansas, Kentucky, Louisiana, Maine, Maryland, Michigan, Minnesota, Mississippi, Missouri, Nevada, New Jersey, New Mexico, New York, North Carolina, Ohio, Oklahoma, Oregon, Pennsylvania, South Carolina, Tennessee, Texas, Virginia, Washington, and West Virginia.

^§^ P-value from chi square test.

**^¶^** Other race = non-Hispanic Asian or Pacific Islander, American Indian or Native Alaskan, and multiracial.

** Other health insurance coverage categories included self-pay and workers’ compensation.

**TABLE 2:** Most common diagnoses and symptoms potentially related to COVID-19* among post–COVID-19 patients and control patients^†^ receiving care in outpatient rehabilitation clinics — United States,**^§^** January 2020–March 2021

Diagnoses^¶^ (ICD-10 code)	No. (%)	
	Post–COVID-19 patients (n = 1,295)	Control patients (n = 2,395)
**Most common diagnoses**		
Neoplasms (C code 189.0; D code I97.2)	17 (1.3)	2,767 (100)
Muscle weakness (generalized), malaise and fatigue (M62.81, R53.0, R53.1, R53.8)	941 (72.7)	1,014 (42.3)
COVID-19 (G93.3, U07.1, Z86.19)	970 (74.9)	12 (0.5)
**Symptoms potentially related to COVID-19**		
Muscle weakness (generalized), malaise and fatigue (M62.81, R53, R53.1, R53.8, R53.81, R53.83)	894 (69.0)	1,430 (59.7)
Muscle weakness (generalized) (M62.81)	572 (44.2)	929 (38.8)
Malaise and fatigue (R53, 53.1, R53.8, R53.81, R53.83)	522 (40.4)	566 (23.6)
Abnormalities of gait and mobility (R26.2, R26.89)	266 (20.5)	205 (8.6)
Acute respiratory failure with hypoxia (J96.01)	26 (2.0)	0 (—)

**Abbreviation:** ICD-10 = *International Classification of Diseases, Tenth Revision.*

* ICD-10 codes at first evaluation in outpatient rehabilitation clinic.

**^†^** Post–COVID-19 patients were defined as those who were referred for post–COVID-19 care to Select Medical’s Recovery and Reconditioning program. In addition, patient history of COVID-19 was assessed by validating whether a patient had either 1) an ICD-10 code for COVID-19 or 2) clinical notes documenting COVID-19 history. Control patients were defined as patients referred for cancer rehabilitation and confirmed with no history of COVID-19 diagnoses by ICD-10 code in the same network and time frame.

^§^ Select Medical’s outpatient rehabilitation clinics are located in Alabama, Alaska, Arizona, California, Colorado, Connecticut, Delaware, Florida, Georgia, Illinois, Indiana, Iowa, Kansas, Kentucky, Louisiana, Maine, Maryland, Michigan, Minnesota, Mississippi, Missouri, Nevada, New Jersey, New Mexico, New York, North Carolina, Ohio, Oklahoma, Oregon, Pennsylvania, South Carolina, Tennessee, Texas, Virginia, Washington, and West Virginia.

^¶^ This list is not exhaustive and is based on nonmutually exclusive ICD-10 codes.

Compared with control patients, post–COVID-19 patients had higher prevalences of reported fair or poor general health (32.9% versus 25.4%), poorer physical health (44.1% versus 32.6%), pain level ≥7 (on a scale of 0–10) (40.4% versus 24.8%), and difficulty with physical activities (32.3% versus 24.2%) (Table 3). Post–COVID-19 patients also reported a higher prevalence of fair or poor overall mental health than control patients (19.1% versus 15.3%). Post–COVID-19 patients and control patients reported more challenges with applied cognition as indicated by T-scores (42.2 versus 41.2), both approximately one SD below the normative sample with which the scale was developed. Post–COVID-19 patients also demonstrated reduced physical endurance on the 6MWT compared with control patients (distance of 303 m versus 377 m; p<0.001) and reported increased difficulty completing chores (38.2% versus 25.2%), navigating stairs (40.2% versus 18.3%), running errands or shopping (34.3% versus 16.0%), and walking for 15 minutes (38.2% versus 16.6%). Compared with control patients, post–COVID-19 patients also reported more difficulty doing usual work or work at home (37.2% versus 20.4%) and challenges in ability to participate in activities with friends (33.0% versus 18.8%). For measures of health care use, post–COVID-19 patients required significantly more visits (median = 9, interquartile range [IQR] = 4–20) than control patients (median = 5, IQR 1–11; p<0.001) and longer therapy duration (median = 35 days, IQR = 15–71 days versus median = 27 days, IQR = 0–57 days; p<0.001).

**TABLE 3:** Measures of mental and physical health, functioning, and treatment among post–COVID-19 patients and control patients* — United States,^†^ January 2020–March 2021

Characteristic	% (95% CI)		
	Post–COVID-19 patients	Control patients	aOR^§^
**General health fair or poor** ^¶^	32.9 (28.8 to 36.9)	25.4 (23.6 to 27.1)	1.64 (1.32 to 2.04)
Mental health**			
Quality of life, fair or poor	19.9 (16.5 to 23.4)	19.3 (17.7 to 20.9)	1.17 (0.91 to 1.50)
Mental health, fair or poor	19.1 (15.7 to 22.6)	15.3 (13.9 to 16.8)	1.34 (1.04 to 1.73)
Satisfaction with social activities, fair or poor	17.4 (14.1 to 20.7)	19.2 (17.6 to 20.7)	0.98 (0.76 to 1.27)
Emotional problems, often or always	12.8 (9.9 to 15.7)	15.0 (13.6 to 16.5)	0.91 (0.68 to 1.22)
Physical health**			
Physical health, fair or poor	44.1 (39.8 to 48.4)	32.6 (30.7 to 34.4)	1.76 (1.43 to 2.15)
Physical activities, little or none at all	32.3 (28.3 to 36.3)	24.2 (22.5 to 25.9)	1.64 (1.32 to 2.03)
Pain, ≥7	40.4 (36.2 to 44.7)	24.8 (23.1 to 26.5)	2.30 (1.86 to 2.83)
Fatigue, severe or very severe	15.7 (12.5 to 18.8)	14.1 (12.7 to 15.5)	1.03 (0.79 to 1.36)
**Physical functional status (with much difficulty or unable to do)^††^**			
Able to do chores such as vacuuming or yard work	38.2 (28.6 to 47.8)	25.2 (23.0 to 27.4)	2.17 (1.42 to 3.35)
Able to go up and down stairs at a normal pace	40.2 (30.5 to 49.9)	18.3 (16.4 to 20.3)	4.12 (2.62 to 6.48)
Able to go for a walk of at least 15 minutes	38.2 (28.6 to 47.8)	16.6 (14.7 to 18.5)	4.60 (2.90 to 7.30)
Able to run errands and shop	34.3 (24.9 to 43.7)	16.0 (14.1 to 17.9)	3.43 (2.17 to 5.42)
**Social participation ability (usually or always)^§§^**			
Trouble doing all of my regular leisure activities with others	22.3 (13.8 to 30.9)	17.3 (15.3 to 19.2)	1.48 (0.88 to 2.50)
Trouble doing all of the family activities that I want to do	23.4 (14.7 to 32.1)	17.4 (15.5 to 19.3)	1.52 (0.91 to 2.54)
Trouble doing all of my usual work (include work at home)	37.2 (27.3 to 47.2)	20.4 (18.3 to 22.4)	2.43 (1.54 to 3.84)
Trouble doing all of the activities with friends that I want to do	33.0 (23.3 to 42.7)	18.8 (16.8 to 20.8)	2.27 (1.41 to 3.64)
**Applied cognition (often or very often)^¶¶^**			
Have to read something several times to understand it	15.7 (11.6 to 19.9)	20.3 (9.8 to 30.9)	0.73 (0.36 to 1.52)
Trouble keeping track of what I was doing if I was interrupted	20.1 (15.5 to 24.6)	18.6 (8.4 to 28.9)	1.09 (0.52 to 2.26)
Difficulty doing more than one thing at a time	22.7 (18.0 to 27.5)	23.7 (12.5 to 34.9)	0.91 (0.46 to 1.80)
Trouble remembering new information, like phone numbers or simple instructions	17.4 (13.1 to 21.7)	18.6 (8.4 to 28.9)	1.12 (0.53 to 2.35)
Trouble thinking clearly	18.7 (14.3 to 23.2)	16.9 (7.1 to 26.8)	1.04 (0.49 to 2.24)
Thinking was slow	18.4 (14.0 to 22.8)	20.3 (9.8 to 30.9)	0.86 (0.42 to 1.77)
Have to work really hard to pay attention or I would make a mistake	20.1 (15.5 to 24.6)	16.9 (7.1 to 26.8)	1.23 (0.58 to 2.62)
Trouble concentrating	20.1 (15.5 to 24.6)	20.3 (9.8 to 30.9)	0.90 (0.44 to 1.83)
Summary scale T-score,*** **mean SD, mean difference**			
Mental health	46.7 (47.2 to 48.7)	47.6 (48.4 to 49.1)	−0.96 (−1.83 to −0.09)
Physical health	40.6 (40.0 to 41.2)	43.8 (43.4 to 44.2)	−3.54 (−4.40 to −2.67)
Physical functional status	37.1 (35.4 to 38.8)	43.5 (43.0 to 44.0)	−7.43 (−9.37 to −5.50)
Social participation ability	52.6 (45.6 to 59.7)	53.0 (51.8 to 54.2)	−0.53 (−5.72 to 4.67)
Applied cognition	42.2 (41.1 to 43.4)	41.2 (38.5 to 43.8)	1.23 (−1.64 to 4.11)
**Physical endurance,^††† ^mean IQR, mean difference**			
6-minute walk test, meters	303.0 (276.6 to 329.4)	377.4 (360.3 to 394.5)	−94.21 (−124.92 to −63.51)
**Health care use, median (IQR) and p-value**			
Days in therapy	35 (15 to 71)	27 (0 to 57)	<0.001
Total number of visits	9 (4 to 20)	5 (1 to 11)	<0.001

**Abbreviations:** aOR = adjusted odds ratio; CI = confidence interval; ICD-10 = *International Classification of Diseases, Tenth Revision*; IQR = interquartile range; Neuro-QoL = Quality of Life in Neurologic Disorders; PROMIS = Patient Reported Outcomes Measurement Information System; SD = standard deviation.

* Post–COVID-19 patients were defined as those who were referred for post–COVID-19 care to Select Medical’s Recovery and Reconditioning program. In addition, patient history of COVID-19 was assessed by validating whether a patient had either 1) an ICD-10 code for COVID-19 or 2) clinical notes documenting COVID-19 history. Control patients were defined as patients referred for cancer rehabilitation and confirmed with no history of COVID-19 diagnoses by ICD-10 code in the same network and time frame.

^†^ Select Medical’s outpatient rehabilitation clinics are located in Alabama, Alaska, Arizona, California, Colorado, Connecticut, Delaware, Florida, Georgia, Illinois, Indiana, Iowa, Kansas, Kentucky, Louisiana, Maine, Maryland, Michigan, Minnesota, Mississippi, Missouri, Nevada, New Jersey, New Mexico, New York, North Carolina, Ohio, Oklahoma, Oregon, Pennsylvania, South Carolina, Tennessee, Texas, Virginia, Washington, and West Virginia.

^§^ Adjusted for age (years, continuous) and sex.

^¶^ Proportions of patients reporting “fair” or “poor” general health.

** Mental and physical health were assessed with PROMIS Scale v1.2 – Global Health (National Institutes of Health). PROMIS items all use a Likert-type response scale. Most questions ask about a person's experience “in general,” with items on fatigue, pain, and emotional problems referencing the past 7 days. The PROMIS global mental health scale includes four items that rate overall mental health (quality of life, mental health, emotional distress, and social activities and roles). The PROMIS global physical health scale includes four items that rate overall physical health (physical functioning, physical activities, pain, and fatigue). Proportions of patients reporting “fair” or “poor” health were calculated for each measure, with the exceptions of emotional problems, physical activities, pain, and fatigue. Proportions of patients reporting “often” or “always” were calculated for emotional problems; “little” or “none at all” for physical activities; and “severe” or “very severe” for fatigue. Pain was measured using a scale of 0–10 and the proportion of patients reporting ≥7 was calculated.

^††^ Physical functional status was assessed with PROMIS Item Bank v2.0 – Physical Function–Short Form 4a. Proportions of patients reporting “with much difficulty” or “unable to do with much difficulty” were calculated for each measure.

**^§§^** Social participation ability was assessed with PROMIS Item Bank v2.0 – Ability to Participate in Social Roles and Activities–Short Form 4a. Proportions of patients reporting “usually” or “always” were calculated for each measure.

**^¶¶^** Applied cognition was assessed with Neuro-QOL Item Bank v1.0 – Applied Cognition – General Concerns (AC-GC)–Short Form. Neuro-QoL AC-GC assesses perceived difficulties in everyday cognitive abilities such as memory, attention, and decision-making. Proportions of patients reporting “often (once a day)” or “very often (several times a day)” were calculated for each measure.

*** Total raw scores were computed by summing items scores that range from 1 to 5, such that higher scores reflect better functioning and are then rescaled to a mean of 50 and SD of 10 using nationally normative data from the U.S. general population.

^†††^ Physical endurance was assessed using the 6-minute walk test. A poor 6-minute walk distance (e.g., <300 m) might have prognostic value (i.e., usually associated with an increased risk of mortality), and a change of 14.0 to 30.5 m might be clinically relevant.

## Discussion

Among patients referred to Select Medical’s outpatient rehabilitation clinics during January 2020–March 2021 (during the COVID-19 pandemic), patients who previously had COVID-19 reported poorer general, mental, and physical health (i.e., overall physical health, physical activities, and pain), and functioning (i.e., physical and social, such as ability to do chores, usual work, or activities with friends) compared with patients with no previous diagnosis of COVID-19 referred for cancer rehabilitation. Also, post–COVID-19 patients did not perform as well as control patients on a measured assessment of physical functioning (6MWT). Finally, post–COVID-19 patients used more rehabilitative services than control patients. These findings indicate that among patients referred to outpatient rehabilitation, those recovering from COVID-19 might have poorer physical health and functional status than do patients with cancer but not COVID-19 and could benefit from additional clinical support, including tailored physical and mental health rehabilitation services.

The identification of poorer physical health among post–COVID-19 patients is consistent with a previous study that found that 92% of post–COVID-19 patients had diagnoses potentially related to post–COVID-19 conditions, including weakness, malaise, fatigue, respiratory failure with hypoxia, and gait abnormalities (*2*,*3*). Poorer self-reported physical and mental health is associated with long-term negative health outcomes including chronic diseases (e.g., diabetes and cardiovascular disease), functional decline (*4*), and mortality (*5*). The lower scores on applied cognitive ability tasks suggest more subtle deficits in cognitive functioning, which might indicate the need for further evaluation and additional need for health care resources and services (*6*). Further, physical function, as measured by the 6MWT, has been shown to be an important outcome for assessing impact of COVID-19 (*4*). Additional studies have shown that patients recovering from COVID-19 have higher incidences of negative health outcomes, including poorer physical health and functional status, and might need additional clinical support such as tailored physical and mental health rehabilitation services (*7*,*8*). These findings have implications for health care systems during and after the COVID-19 pandemic (*9*). Postacute sequalae associated with COVID-19 have not been comprehensively described, and data from studies of long-term follow-up to provide reliable estimates of the long-term sequelae associated with COVID-19 are still emerging (*6*–*8*). Continued assessments of self-reported health data are important to characterize the sequelae of novel infectious diseases and are critical for developing cost-effectiveness estimates for lifesaving interventions, such as vaccines and other potentially important rehabilitation therapies and interventions, including physical therapy, occupational therapy, and services and therapies associated with cognitive and functional decline (*9*,*10*).

The findings in this report are subject to at least six limitations. First, date of infection was not available; therefore, time-varying effects associated with infection date could not be examined. Second, data on severity of illness, including hospitalization status, were not available, precluding assessment of the impact of illness severity on post–COVID-19 conditions. Third, given the large amount of missing data (>50%) for many demographic variables (e.g., race, ethnicity, employment status, and occupation), which are common limitations in large EHR data sets, it was not possible to control for additional demographic differences. Fourth, the absence of pre–COVID-19 assessments did not permit controlling for premorbid function. Fifth, the types of cancer diagnoses and treatments were not available, which is an important consideration given heterogeneity of cancer sequelae. Similarly, assessing other comorbidities was not possible; post–COVID-19 patients might have had more underlying medical conditions (e.g., diabetes or obesity) than did control patients, which could explain poorer physical and mental health measures. However, given that patients in the post–COVID-19 group were younger and more commonly employed than were those in the control group, it is likely that these two populations are different with regard to demographic factors and the prevalence of comorbid chronic conditions. Finally, referral to physical rehabilitation depended on nonstandardized clinical judgment, which might have led to differences in patient population by group. Therefore, these results should not be interpreted to mean that post–COVID-19 patients overall had poorer physical and mental health than patients with cancer. Instead, results indicate that post–COVID-19 patients specifically referred to a large physical rehabilitation network had poorer health measures than those referred for cancer, which indicates that some patients recovering from COVID-19 had substantial rehabilitation needs.

Patients recovering from COVID-19 might experience continued poor health and could benefit from additional support and tailored physical and mental health rehabilitation services. Health care systems and providers should be prepared to recognize and meet the ongoing needs of this patient population. Efforts to increase COVID-19 vaccination could include messaging that states that preventing COVID-19 also prevents post–COVID-19 conditions with potential effects on long-term health.

**Summary**

**What is already known about this topic?**

COVID-19 patients might experience symptoms that persist months after initial infection.

**What is added by this report?**

Compared with control patients enrolled in a cancer rehabilitation program, adult post–COVID-19 patients referred for rehabilitation services reported poorer physical health and being less able to engage in physical activities and activities of daily living. Patients recovering from COVID-19 also had significantly higher health care use than control patients.

**What are the implications for public health practice?**

Patients recovering from COVID-19 might require tailored physical and mental health rehabilitation services.

## Footnotes

* Data used were from Select Medical, a network of rehabilitation clinics in 36 states and the District of Columbia. https://www.selectmedical.com/

†
https://www.thoracic.org/statements/resources/pfet/sixminute.pdf

§ *International Classification of Diseases, Tenth Revision* codes used to examine potential post-COVID condition were J96.01, M62.81, R.26.2, R26.89 R53, R53.1, and R53.83.

¶ PROMIS items use a Likert-type response scale (https://commonfund.nih.gov/promis/index). The 10 PROMIS items used in this analysis included overall self-rated health; overall quality of life; overall physical health; overall mental health; and individual items on fatigue, pain, emotional distress, and social activities and roles. Most questions asked about a person's experience “in general,” with items on fatigue, pain, and emotional problems experienced during the past 7 days. Psychometric evaluation of the PROMIS global health items were based on two global physical health (GPH) and global mental health (GMH) scales. The PROMIS GPH scale included four items that rated overall physical health (physical functioning, physical activities, pain, and fatigue). GPH and GMH total raw scores were computed by summing item scores that ranged from 1 to 5, such that higher scores reflected better functioning and are then rescaled to a mean of 50 and an SD of 10 using nationally normative data from the U.S. general population. The estimated correlation between the GPH and GMH was 0.63.

** Neuro-QoL is a set of self-report measures that assesses the health-related quality of life of adults with neurologic disorders. Neuro-QoL AC-GC assesses perceived difficulties in everyday cognitive abilities, such as memory, attention, and decision-making. https://www.healthmeasures.net/explore-measurement-systems/neuro-qol

†† Physical endurance was assessed using the 6-minute walk test. A poor 6-minute walk distance (e.g., <300 m) might have prognostic value (i.e., usually associated with an increased risk of mortality), and a change of 14.0 to 30.5 m might be clinically relevant.

§§ Other demographic variables besides sex and age had substantial proportions of missing data (26%–75%); therefore, these variables were not included in the analysis.

¶¶ 45 C.F.R. part 46, 21 C.F.R. part 56; 42 U.S.C. Sect. 241(d); 5 U.S.C. Sect. 552a; 44 U.S.C. Sect. 3501 et seq.
