# *Erratum*: Vol. 70, No. 27

**DOI:** 10.15585/mmwr.mm7033a5

**Published:** 2021-08-20

In the report “Outcomes Among Patients Referred to Outpatient Rehabilitation Clinics After COVID-19 diagnosis — United States, January 2020–March 2021,” on page 967, the following authors’ names should have read, “Meredith **G. Dixon**, MD” and “Caitlyn **Lutfy**, MPH.” On page 970, in Table 3, in the row for “Social participation ability,” in the columns for “Post–COVID-19 patients,” “Control patients,” and “mean difference” the summary scale T-score mean standard deviations and mean differences should have read, “**46.6 (44.7 to 48.6)**,” “**50.5 (50.0 to 51.1)**,” and “** −4.2 (−6.4 to −2.0)**,” respectively. 

